# Prise en charge diététique du diabète gestationnel: efficacité et adhésion aux recommandations diététiques, étude transversale comparative entre les secteurs public et privé de la santé dans la région de Marrakech-Safi, Maroc

**DOI:** 10.11604/pamj.2025.52.138.47565

**Published:** 2025-12-04

**Authors:** Hafssa Abdellaoui, Nadia Ouzennou, Samia Rkha

**Affiliations:** 1 Laboratoire de Pharmacologie, Neurobiologie, Anthropologie, Environnement et Comportement, Faculté des Sciences Semlalia, Université Cadi Ayyad, Marrakech, Maroc,; 2 Institut Supérieur des Professions Infirmières et Techniques de Santé, Marrakech, Maroc

**Keywords:** Diabète gestationnel, secteur public, secteur privé, observance, Marrakech-Safi, Gestational diabetes, public sector, private sector, adherence, Marrakech-Safi

## Abstract

**Introduction:**

le diabète gestationnel (DG) est une complication fréquente de la grossesse, associée à des risques pour la mère et l'enfant. Sa prise en charge repose sur l'adhésion aux recommandations diététiques dont l'efficacité peut varier selon le contexte socio-économique et le type de système de santé. Au Maroc, peu d'études ont comparé les secteurs public et privé.

**Méthodes:**

une étude transversale rétrospective a porté sur 331 femmes atteintes de DG dans la région de Marrakech-Safi (266 dans le secteur public, 65 dans le secteur privé). Les données ont été recueillies par questionnaire standardisé. Une régression logistique multivariée a identifié les facteurs associés aux différences de prise en charge et d'efficacité. Les résultats sont présentés en ORa, IC 95% et valeurs p.

**Résultats:**

un revenu familial supérieur au SMIG (ORa: 8,99; IC 95% 1,92-42,13; p= 0,005) et la couverture sociale (ORa: 25,5; IC 95% 5,06-128,6; p< 0,001) étaient associés au suivi dans le secteur privé. L'adhésion diététique était plus élevée chez les femmes du privé (ORa: 2,08; IC 95% 1,04-4,19; p= 0,040). L'activité physique était favorisée par le suivi privé (ORa: 2,18; IC 95% 1,18-4,04; p = 0,013). Le contrôle glycémique était meilleur dans le privé (ORa: 0,20; IC 95% 0,09-0,43; p< 0,001). Le suivi médical régulier était plus fréquent dans le privé (ORa: 0,33; IC 95% 0,12-0,91; p= 0,031).

**Conclusion:**

l'étude révèle d'importantes disparités entre les secteurs public et privé dans la prise en charge du DG, influencées par les ressources économiques. Le renforcement du secteur public apparaît nécessaire pour réduire ces inégalités.

## Abstract (en)

**Introduction:**

gestational diabetes is a frequent complication of pregnancy, associated with risks for both mother and child. Its management relies on adherence to dietary recommendations, the effectiveness of which may vary according to the socio-economic context and the type of healthcare system. In Morocco, few studies have compared the public and private sectors.

**Methods:**

a retrospective cross-sectional study included 331 women with GDM in the Marrakech-Safi region (266 public, 65 private). Data were collected using a standardised questionnaire. Multivariable logistic regression was performed to identify factors associated with management and therapeutic outcomes. Results are presented as adjusted odds ratios (aOR), 95% confidence intervals, and p-values.

**Results:**

family income above the minimum wage (aOR: 8.99, 95% CI 1.92-42.13; p= 0.005) and social security coverage (aOR: 25.5, 95% CI 5.06-128.6; p< 0.001) were associated with follow-up in the private sector. Dietary adherence was higher among women in the private sector (aOR: 2.08, 95% CI 1.04-4.19; p= 0.040). Physical activity was favoured by private follow-up (aOR: 2.18, 95% CI 1.18-4.04; p= 0.013). Glycemic control was better in the private sector (aOR: 0.20, 95% CI 0.09-0.43; p< 0.001). Regular medical follow-up was more frequent in the private sector (aOR: 0.33, 95% CI 0.12-0.91; p= 0.031).

**Conclusion:**

this study reveals marked disparities between public and private sectors in the management of GDM in Morocco, largely influenced by economic resources. Strengthening public sector capacity is necessary to enhance the equity and efficiency of care.

## Introduction

Le diabète gestationnel est un trouble de l'intolérance au glucose qui survient pendant la grossesse chez des femmes. Il a des implications maternelles et fœtales majeures, augmentant le risque de complications périnatales telles que la macrosomie, la pré-éclampsie et le diabète de type 2 à long terme pour la mère [1-4]. La prise en charge repose principalement sur des modifications strictes du régime alimentaire et une surveillance continue de la glycémie, l'insulinothérapie n'étant envisagée que si le régime alimentaire est insuffisant [5-8]. Une intervention nutritionnelle appropriée est donc essentielle pour maintenir la glycémie maternelle dans une fourchette sûre. Les pratiques de gestion varient en fonction de la géographie et de la structure du système de santé. Des études mettent en évidence des différences entre les milieux ruraux et urbains [9,10], ainsi qu'entre les systèmes de santé publics et privés. Au Maroc, ces disparités sont marquées: les femmes en milieu rural ont un accès limité aux spécialistes et font principalement appel aux sages-femmes [11-14], alors que les patientes en milieu urbain sont plus souvent suivies par des endocrinologues et reçoivent des conseils diététiques [15]. Le système de santé marocain est divisé en secteurs publics et privés. Les établissements publics comme les hôpitaux et centres de santé gérés par le ministère de la santé et d'autres départements desservent principalement les populations rurales et à faibles revenus, tandis que les soins privés, généralement plus onéreux, sont principalement accessibles aux femmes urbaines [16].

Pour faire face à l'augmentation de la prévalence du diabète, y compris du DG, le Maroc a mis en œuvre un plan national de prévention et de prise en charge (2012-2016) aligné sur les lignes directrices de l'Organisation mondiale de la Santé, visant à réduire les complications grâce à des campagnes de sensibilisation [17]. Une formation spécifique pour les médecins généralistes, les sages-femmes et les infirmières du secteur public a été mise en place pour pallier la pénurie de spécialistes, en mettant l'accent sur le dépistage précoce et la prise en charge multidisciplinaire [18,19]. Le dépistage s'effectue généralement au moyen d'un premier test de glycémie suivi, si nécessaire, d'un test de confirmation ou d'un seul test de tolérance au glucose par voie orale. Une fois le diagnostic posé, des équipes multidisciplinaires (sages-femmes, gynécologues, endocrinologues, nutritionnistes) fournissent des conseils sur le régime alimentaire, l'activité physique et l'auto-surveillance glycémique [19]. Au Maroc, les soins de santé publics sont caractérisés par des protocoles standardisés et une accessibilité, mais aussi par des ressources limitées, avec une prise en charge souvent assurée par des médecins généralistes ou des sages-femmes [20]. À l'inverse, les soins privés, soutenus par des moyens financiers plus importants, offrent un suivi plus spécialisé, notamment par des endocrinologues et des diabétologues [21]. Cette étude a pour objectif de comparer la prise en charge diététique du diabète gestationnel entre les secteurs public et privé de la région de Marrakech-Safi, en se focalisant sur l'adhésion aux recommandations diététiques et leur efficacité.

## Méthodes

**Conception et cadre de l'étude:** cette étude transversale rétrospective a été réalisée entre octobre 2021 et janvier 2023. Les participantes ont été recrutées dans les établissements de santé publics et privés situés dans la région de Marrakech-Safi.

**Population de l'étude:** les critères d'inclusion comprenaient une confirmation diagnostique de diabète gestationnel par le personnel de santé (sages-femmes, médecin généraliste ou spécialiste). Les critères d'exclusion concernaient les femmes atteintes de diabète de type 1 ou de type 2 diagnostiqué avant la grossesse. La population cible dans la zone de recrutement a été estimée à 8554 femmes enceintes. En utilisant une prévalence supposée de 16% pour le diabète gestationnel, la taille minimale requise de l'échantillon a été calculée à 301, et 331 femmes ont finalement été incluses.

**Collecte des données:** les données ont été obtenues à l'aide d'un questionnaire structuré administré lors des consultations prénatales. Ce questionnaire portait sur les caractéristiques sociodémographiques et économiques, la prise en charge diététique, la fréquence des consultations, les habitudes alimentaires (y compris le grignotage), le type de prestataire de soins, l'activité physique et la surveillance de la glycémie. Les données ont été recueillies par le premier auteur dans des établissements publics et privés de la région de Marrakech-Safi.

**Définitions:** l'adhésion aux recommandations diététiques a été définie en trois niveaux: bon (toujours/souvent conforme aux recommandations sans écarts), modéré (conformité partielle avec 1-10 écarts hebdomadaires), et mauvais (rare/jamais conforme avec >10 écarts). L'efficacité du régime alimentaire a été définie comme une amélioration de la glycémie, mesurée par une normalisation ou une réduction significative des niveaux.

**Analyse statistique:** les données ont été analysées à l'aide de SPSS version 18. Des statistiques descriptives ont été utilisées pour résumer les variables catégorielles et continues. Les comparaisons de groupe entre les secteurs public et privé ont été effectuées à l'aide de tests du chi carré pour les variables catégorielles et du test de Mann-Whitney pour les variables continues distribuées de manière non normale. Une régression logistique univariée a d'abord été effectuée pour évaluer les associations entre chaque variable indépendante et les résultats des soins. Les variables ayant un P<.20 dans les analyses univariées, ainsi que celles considérées comme cliniquement pertinentes, ont été introduites dans des modèles de régression logistique multivariables. Les modèles finaux ont été ajustés pour tenir compte des facteurs de confusion potentiels, notamment l'âge, le secteur des soins de santé et le statut d'assurance. Les résultats ont été exprimés sous forme de rapports de cotes ajustés (ORa) avec des intervalles de confiance à 95%, et la signification statistique a été fixée à P<.05.

**Considérations éthiques:** l'étude a été approuvée par la direction régionale de la santé de Marrakech-Safi (autorisation n° 3235, datée du 10/05/2021). Les délégués locaux à la santé ont été informés pour coordonner les activités de l'enquête. Avant de participer, toutes les femmes ont reçu des informations claires sur les objectifs et les procédures de l'étude, et un consentement éclairé oral a été obtenu. La confidentialité et l'anonymat des données ont été strictement respectés.

## Résultats

**Figure 1:**
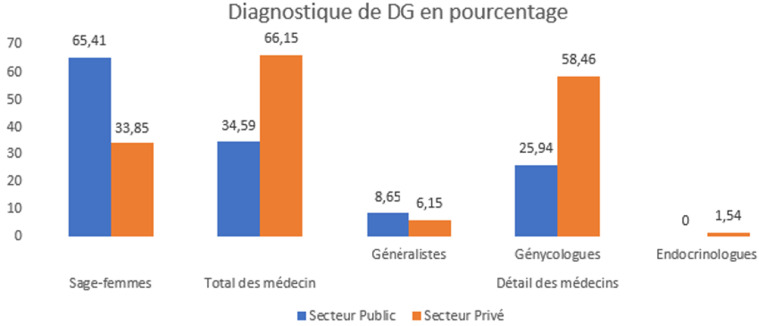
diagnostic du diabète gestationnel: comparaison entre secteurs public et privé selon le type de professionnel de santé

**Figure 2:**
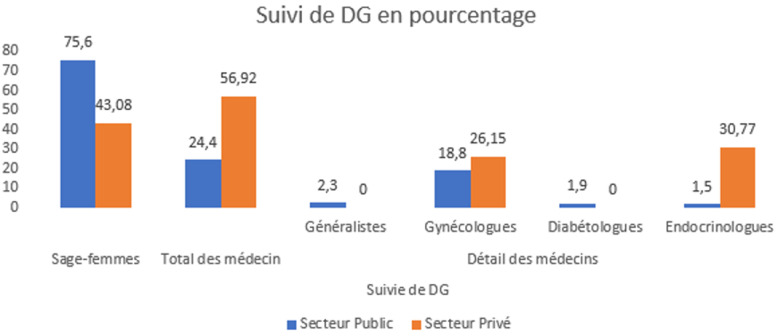
répartition des professionnels de santé impliqués dans le suivi du diabète gestationnel selon le secteur

L'âge moyen était similaire entre les groupes (27,0 contre 27,4 ans). Dans le secteur public, les femmes étaient principalement rurales (55,3%), avec des revenus faibles (59,0%), un niveau d'éducation limité (18,8% d'analphabètes) et surtout des femmes au foyer (94,7%). En revanche, les femmes du secteur privé étaient majoritairement urbaines (80%), toutes alphabétisées, plus riches (78,5%) et plus souvent employées (27,7%) (Tableau 1). Le diagnostic était majoritairement assuré par des sages-femmes dans le secteur public (65,4%), et par des médecins dans le secteur privé (66,2%; X^2^= 21,6; P<,001) (Figure 1). Le suivi était également majoritairement assuré par des sages-femmes dans le secteur public (75,6%) contre des médecins dans le secteur privé (56,9%; X^2^= 25,9; P<,001) (Figure 2).

**Tableau 1:** profil démographique et socio-économique des femmes atteintes de diabète gestationnel selon le secteur de suivi

Variable	Modalité	Secteur du suivi			
		Public		Privé	
		N	%	N	%
**Age moyen (ans)**	-	27,01		27,43	
**Lieu d'habitat**	Urbain Rural	119 147	44,74 55,26	52 13	80 20
**Analphabétisme de la femme**	Alphabète Analphabète	216 50	81,20 18,80	65 0	100 0,0
**Profession de la Femme**	Femme au foyer Femme avec profession	252 14	94,74 5,26	47 18	72,31 27,69
**Revenu mensuel de la famille**	Inférieur au SMIG Supérieur au SMIG	157 109	59,02 40,98	14 51	21,54 78,46
**Couverture sociale**	Oui Non	194 72	72,93 27,07	44 21	67,69 32,31

N: Effectif; % Pourcentage; SMIG: Salaire Minimum Interprofessionnel Garanti = 3100 DH; DH: Dirham Marocain = 0,10 USD

**Figure 3:**
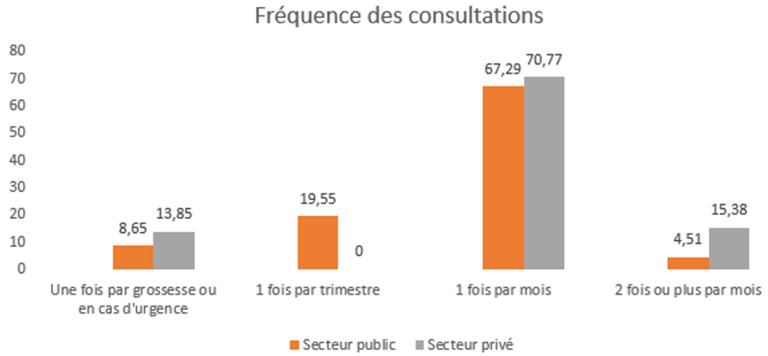
fréquence des consultations prénatales des femmes atteintes de diabète gestationnel selon le secteur de suivi

Les prescriptions diététiques étaient très contrastées: les sages-femmes dominaient dans le secteur public (75,2%) et les médecins dans le secteur privé (61,5%). L'absence de prescription était plus fréquente dans le secteur public (21,8% contre 7,7%; X^2^= 144,4; P<,001). Le régime alimentaire restait le traitement principal, plus fréquent dans le secteur privé (96,9% contre 77,1%). L'absence de traitement n'a été rapportée que dans le secteur public (18,4%; X^2^= 15,5; P=,001). L'activité physique est plus fréquente dans le secteur public (65,8% contre 44,6%; X^2^= 9,9; P=,002), tandis que la surveillance régulière de la glycémie est plus élevée dans le secteur privé (84,6% contre 49,2%; X^2^ = 26,5; P<,001). Le suivi de la grossesse était également plus fréquent dans le secteur privé (86,2% contre 75,6%; X^2^= 3,4; P=,026) (Tableau 2, Figure 3).

**Tableau 2:** comparaison des pratiques de prise en charge du diabète gestationnel entre le secteur public et le secteur privé

Variable	Modalité	Secteur du suivi		P-value
		Public N (%)	Privé N (%)	
**Personnel prescrivant le régime alimentaire**	Sage-femme Médecins Aucun	200 (75,2) 8 (3) 58 (21,8)	20 (30,8) 40 (61,5) 5 (7,7)	P<,001
**Traitement prescrit**	Médicament Régime alimentaire Aucun	12 (4,5) 205 (77,1) 49 (18,4)	2 (3,1) 63 (96,9) 0 (0)	P=,001
**Activité physique**	Oui Non	175 (65,8) 91 (34,2)	29 (44,6) 36 (55,4)	P=,002
**Contrôle de la glycémie**	Oui Non	131 (49,2) 135 (50,8)	55 (84,6) 10 (15,4)	P<,001
**Surveillance de la grossesse**	Oui Non	201 (75,6) 65 (24,4)	56 (86,2) 9 (13,8)	P=,026

N: effectif; % pourcentage

**Figure 4:**
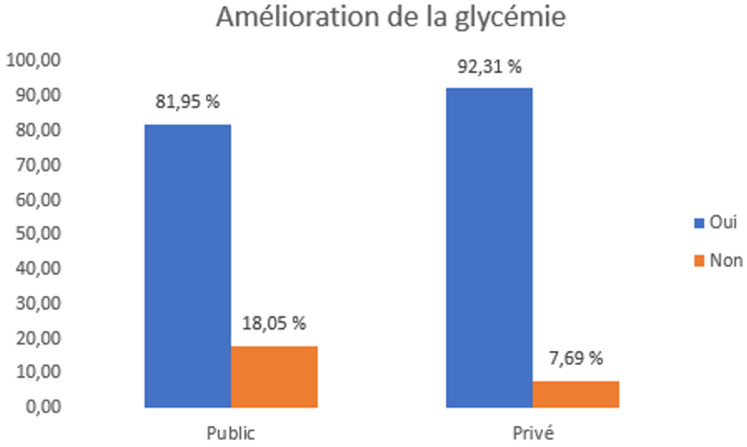
amélioration de la glycémie chez les femmes atteintes de diabète gestationnel selon le secteur de suivi

Les femmes du secteur privé ont plus souvent déclaré suivre toujours/souvent les recommandations alimentaires (89,2% contre 67,7%; X^2^= 15,7; P=,003). Les aliments sucrés prédominaient dans le secteur public (33,8%), tandis que les aliments salés étaient préférés dans le secteur privé (44,6%; X^2^= 65,7; P<,001). L'adhésion globale était plus élevée dans le groupe privé (58,5% de bonne adhésion contre 35,3%; P<,001) (Tableau 3). Une amélioration glycémique a été observée chez 92,3% des femmes du secteur privé vs 81,9% dans le public (X^2^= 4,16; P=,043). L'absence d'amélioration était plus fréquente dans le public (18,1% contre 7,7%) (Figure 4).

**Tableau 3:** adhésion aux recommandations alimentaires et comportements nutritionnels des femmes atteintes de diabète gestationnel selon le secteur de suivi

Variable	Modalité	Secteur du suivi		P-value
		Public	Privé	
		N (%)	N (%)	
**Suivi des recommandations alimentaires**	Parfois, rarement ou jamais Toujours ou souvent	86 (32,3) 180 (67,7)	7 (10,8) 58 (89,2)	P=,003
**Nombre d'écarts par semaine**	0 écart Entre 1 et 10 écarts Plus de 10 écarts	94 (35,3) 116 (43,6) 56 (21,1)	39 (60) 21 (32,3) 5 (7,7)	P<,001
**Grignotage entre les repas**	Jamais Rarement Souvent Toujours	140 (52,6) 75 (28,2) 31 (11,7) 20 (7,5)	12 (18,5) 44 (67,7) 2 (3,1) 7 (10,7)	P<,001
**Nature des aliments grignotés**	Sucrée Salée Les deux Epicée	90 (33,8) 18 (6,8) 17 (6,4) 5 (1,9)	10 (15,4) 29 (44,6) 6 (9,2) 2 (3)	P<,001
**Adhérence**	Mauvaise Moyenne Bonne	61 (22,9) 111 (41,7) 94 (35,3)	5 (7,7) 22 (33,8) 38 (58,5)	P<,001

N: effectif; %: pourcentage

Le Tableau 4 présente les résultats des régressions logistiques multivariées évaluant la relation entre le secteur de suivi et les différents indicateurs de prise en charge, après ajustement sur les caractéristiques sociodémographiques et cliniques. L'adhésion diététique était significativement associée au suivi dans le secteur privé (ORa: 2,08, IC 95% 1,04-4,19; p= 0,040) et influencée négativement par l'inactivité professionnelle (ORa: 0,19, IC 95% 0,07-0,49; p= 0,001). Pour l'activité physique, le suivi privé (ORa: 2,18, IC 95% 1,18-4,04; p= 0,013) et l'absence de couverture sociale (ORa: 0,50, IC 95% 0,29-0,87; p= 0,013) favorisaient sa pratique. Le contrôle glycémique était meilleur chez les femmes suivies dans le privé (ORa: 0,20, IC 95% 0,09-0,43; p< 0,001), alors que l'inactivité professionnelle augmentait le risque de déséquilibre (ORa= 6,12; IC 95%: 1,72-21,8; p= 0,005). Enfin, le suivi médical régulier était plus fréquent dans le secteur privé (ORa: 0,33, IC 95% 0,12-0,91; p= 0,031) et moins observé chez les femmes à revenu supérieur au SMIG (ORa: 0,52, IC 95% 0,28-0,96; p= 0,035).

**Tableau 4:** analyse multivariée de l’association entre le secteur de suivi (public/privé) et les indicateurs de prise en charge des femmes atteintes de diabète gestationnel

Variable	ORs non ajustés (95% CI)	P-value	ORs ajustés (95% CI)	P-value
**Adhésion**	-	-	-	-
Secteur privé (vs public)	2,58 (1,484,48)	,001	**2,08 (1,044,19)**	**,040**
Âge≥35 ans	0,82 (0,441,50)	,51	0,64 (0,321,27)	,201
Analphabète	0,90 (0,491,66)	,74	0,60 (0,311,17)	,133
Revenu > SMIG	0,44 (0,280,69)	<,001	- (non estimable)	-
Couverture sociale (oui)	0,59 (0,360,99)	,044	0,63 (0,371,09)	,100
Sans emploi	0,16 (0,070,37)	<,001	**0,19 (0,070,49)**	**,001**
**Activité physique**	-	-	-	-
Secteur privé (vs public)	**2,39 (1,384,14)**	,002	**2,18 (1,184,04)**	**,013**
Âge ≥35 ans	1,28 (0,712,33)	,41	1,61 (0,843,09)	,154
Analphabète	**2,19 (1,104,38)**	,026	2,13 (0,994,56)	,051
Revenu > SMIG	**0,61 (0,390,95)**	,030	0,96 (0,571,61)	,880
Couverture sociale (oui)	**0,53 (0,310,89)**	,016	**0,50 (0,290,87)**	,013
Sans emploi	**0,45 (0,210,93)**	,032	0,79 (0,351,82)	,581
**Contrôle glycémique**	-	-	-	-
Secteur (Privé vs Public)	0,18 (0,090,36)	<,001	**0,20 (0,090,43)**	**<,001**
Âge ≥ 35 ans	1,28 (0,712,33)	,41	1,12 (0,582,17)	,72
Analphabétisme	0,62 (0,341,13)	,12	0,85 (0,441,64)	,63
Revenu > SMIG	1,49 (0,962,31)	,07	0,81 (0,491,34)	,41
Couverture sociale (Oui )	1,37 (0,852,22)	,19	1,52 (0,902,58)	,12
Sans emploi	8,74 (2,6129,3)	<,001	**6,12 (1,7221,8)**	,005
**Suivi médical**	-	-	-	-
Secteur de suivi (Privé)	0,30 (0,120,78)	,013	0,33 (0,120,91)	,031
Classe d'âge ≥ 35 ans)	1,89 (0,963,71)	,064	1,87 (0,913,85)	,091
Analphabéétisme	0,62 (0,311,24)	,176	0,73 (0,341,59)	,428
Revenu > SMIG	0,82 (0,471,42)	,476	0,52(0,280,96)	,035
Couverture sociale	0,93 (0,501,73)	,827	1,08 (0,562,06)	,825
Sans emploi	3,85 (0,8916,53)	,070	3,76 (0,8217,18)	,087

Abréviations: OR = Odds ratio; ORa = Odds ratio ajusté; IC = intervalle de confiance; DG = diabète gestationnel; SMIG = salaire minimum interprofessionnel garant

## Discussion

Le régime alimentaire reste la pierre angulaire de la prise en charge du diabète gestationnel, permettant un contrôle glycémique équilibré dans la majorité des cas [22-24]. Cette étude visait à comparer l'observance diététique et son efficacité entre les femmes suivies dans les établissements de santé publics et privés de la région de Marrakech-Safi. Les résultats ont mis en évidence des différences frappantes: les femmes suivies dans le secteur privé ont bénéficié d'une surveillance plus étroite, ont montré une meilleure adhésion aux recommandations et ont obtenu une plus grande amélioration des résultats glycémiques, alors que les femmes du secteur public ont été confrontées à plus de déviations et à une plus faible adhésion.

Les taux d'observance étaient significativement plus élevés dans le secteur privé (58,5% contre 35,3%), avec moins de déviations alimentaires, alors que le manque d'observance était plus fréquent dans le système public. Ces contrastes reflètent probablement des inégalités structurelles: dans le secteur privé, les femmes bénéficient souvent de conseils personnalisés et d'un meilleur accès aux ressources [25], alors que dans le secteur public, des pratiques culturelles telles que les repas en commun, associées à des contraintes financières et éducatives, compromettent une adhésion stricte [26,27]. Il est donc essentiel de s'attaquer à ces obstacles culturels et socio-économiques pour améliorer l'observance.

Les habitudes alimentaires divergent également: les collations sucrées étaient plus fréquentes dans le secteur public, tandis que les aliments salés prédominaient dans le secteur privé. Ces résultats illustrent la manière dont les conditions socio-économiques influencent le comportement alimentaire [28,29]. Dans le secteur privé, des conseils structurés et soutenus par des ressources favorisent des choix plus sains, tandis que les femmes du secteur public, qui ont souvent des moyens limités, luttent pour adopter des pratiques alimentaires optimales. Paradoxalement, les femmes ayant un revenu supérieur au salaire minimum étaient moins susceptibles d'assurer un suivi médical régulier (ORa= 0,52; IC 95%: 0,28-0,96; p= 0,035), ce qui pourrait refléter une tendance à sous-estimer le besoin de soins continus dans les groupes plus aisés [30,31].

L'organisation des soins de santé a également contribué aux disparités. Dans le système public, les sages-femmes assuraient la majeure partie du suivi, garantissant ainsi la continuité, mais avec des ressources limitées. En revanche, les patientes du secteur privé étaient plus souvent suivies par des spécialistes, ce qui contribuait à une surveillance plus étroite et à une meilleure observance [23,32]. De même, l'autocontrôle de la glycémie était beaucoup plus fréquent dans le secteur privé, tandis que l'activité physique était plus souvent rapportée dans le secteur public. Ces comportements complémentaires soulignent l'importance d'une approche holistique associant suivi médical et changement de mode de vie [33,34]. Enfin, la prise en charge diététique était plus efficace dans le secteur privé, avec plus de 90% des patients déclarant une amélioration de leur glycémie, contre environ 82% dans le système public. La forte association observée entre le chômage et un mauvais contrôle glycémique (ORa= 6,12; IC 95%: 1,72-21,8; p= 0,005) souligne la nécessité d'interventions ciblées sur les femmes vulnérables afin d'assurer l'équité des soins [35,36]. Le renforcement de la formation professionnelle, l'amélioration de l'accès aux ressources nutritionnelles et l'harmonisation des pratiques entre les secteurs pourraient contribuer à réduire ces disparités.

Cette étude présente plusieurs points forts, notamment une comparaison innovante entre les systèmes de santé publics et privés, un échantillon représentatif de la région de Marrakech-Safi et l'intégration des facteurs socioculturels dans l'analyse quantitative. Elle présente néanmoins des limites: l'influence potentielle de pratiques culturelles et sociales non mesurées, l'impact du COVID-19 sur l'accès aux soins, l'hétérogénéité des protocoles entre les secteurs et sa restriction à une seule région, qui peut limiter la généralisation.

## Conclusion

Cette étude met en évidence des disparités importantes entre les secteurs public et privé dans la prise en charge du diabète gestationnel dans la région de Marrakech-Safi. Le secteur privé se caractérise par une meilleure adhésion aux recommandations diététiques, une plus grande observance et une plus grande efficacité dans le contrôle glycémique, alors que le secteur public est limité par des contraintes structurelles et socio-économiques. Ces résultats soulignent l'impact direct des inégalités de ressources et d'organisation des soins sur la prise en charge du diabète gestationnel et mettent en évidence la nécessité de combler ces lacunes pour améliorer les résultats maternels dans la région étudiée.

### Etat des connaissances sur le sujet

- Le diabète gestationnel nécessite une prise en charge diététique stricte et une surveillance glycémique continue;
- Les disparités socio-économiques et du système de santé influencent l'accès aux soins maternels et leurs résultats;
- Les sages-femmes jouent souvent un rôle central dans la gestion de la grossesse par le secteur public dans les pays à faibles ressources.

### Contribution de notre étude à la connaissance

- Première étude comparative au Maroc montrant des disparités significatives entre les secteurs public et privé dans la prise en charge diététique du diabète gestationnel;
- Le suivi par le secteur privé garantit une meilleure adhésion aux recommandations et de meilleurs résultats glycémiques par rapport aux soins de santé publics;
- Urgence de renforcer les capacités du secteur public afin de réduire les inégalités et d'améliorer les résultats pour les mères et les nouveau-nés.

## Remerciements

Les auteurs remercient chaleureusement l'ensemble du personnel médical et paramédical des établissements de santé de la région de Marrakech-Safi pour leur collaboration et leur disponibilité tout au long de la collecte des données. Nous exprimons également notre gratitude aux femmes enceintes ayant accepté de participer à cette étude, pour leur confiance et leur précieuse contribution.
